# Identification of Potential MHC Class-II-Restricted Epitopes Derived from *Leishmania donovani* Antigens by Reverse Vaccinology and Evaluation of Their CD4+ T-Cell Responsiveness against Visceral Leishmaniasis

**DOI:** 10.3389/fimmu.2017.01763

**Published:** 2017-12-14

**Authors:** Manas Ranjan Dikhit, Akhilesh Kumar, Sushmita Das, Budheswar Dehury, Ajaya Kumar Rout, Fauzia Jamal, Ganesh Chandra Sahoo, Roshan Kamal Topno, Krishna Pandey, V. N. R. Das, Sanjiva Bimal, Pradeep Das

**Affiliations:** ^1^BioMedical Informatics Division, Rajendra Memorial Research Institute of Medical Sciences, Patna, India; ^2^Department of Immunology, Rajendra Memorial Research Institute of Medical Sciences, Patna, India; ^3^Department of Microbiology, All India Institute of Medical Sciences, Patna, India; ^4^Biomedical Informatics Centre, ICMR-Regional Medical Research Centre, Odisha, India; ^5^Biotechnology Laboratory, ICAR-Central Inland Fisheries Research Institute, Kolkata, India; ^6^Department of Microbiology, Rajendra Memorial Research Institute of Medical Sciences, Patna, India; ^7^Department of Clinical Medicine, Rajendra Memorial Research Institute of Medical Sciences, Patna, India; ^8^Department of Molecular Parasitology, Rajendra Memorial Research Institute of Medical Sciences, Patna, India

**Keywords:** *Leishmania*, CD4 epitope, immunoinformatics, visceral leishmaniasis, IFN-γ, vaccine

## Abstract

Visceral leishmaniasis (VL) is one of the most neglected tropical diseases for which no vaccine exists. In spite of extensive efforts, no successful vaccine is available against this dreadful infectious disease. To support vaccine development, an immunoinformatics approach was applied to screen potential MHC class-II-restricted epitopes that can activate the immune cells. Initially, 37 epitopes derived from six stage-dependent, overexpressed antigens were predicted, which were presented by at least 26 diverse MHC class-II allele. Based on a population coverage analysis and human leukocyte antigen cross-presentation ability, six of the 37 epitopes were selected for further analysis. Stimulation with synthetic peptide alone or as a cocktail triggered intracellular IFN-γ production. Moreover, specific IgG antibodies were detected in the serum of active VL cases against P1, P4, P5, and P6 in order to evaluate the peptide effect on the humoral immune response. Additionally, most of the peptides, except P2, were found to be non-inducers of CD4+ IL-10 against both active VL as well as treated VL subjects. This finding suggests there is no role of these peptides in the pathogenesis of *Leishmania*. Peptide immunogenicity was validated in BALB/c mice immunized with a cocktail of synthetic peptide emulsified in complete Freund’s adjuvant/incomplete Freund’s adjuvant. The immunized splenocytes induced strong spleen cell proliferation upon parasite re-stimulation. Furthermore, increased IFN-γ, interleukin-12, IL-17, and IL-22 production augmented with elevated nitric oxide (NO) synthesis is thought to play a crucial role in macrophage activation. In this investigation, we identified six MHC class-II-restricted epitope hotspots of *Leishmania* antigens that induce CD4+ Th1 and Th17 responses, which could be used to potentiate a human universal T-epitope vaccine against VL.

## Introduction

The different forms of leishmaniasis comprise an imperative group of neglected tropical diseases (1, 2), which influence and impact “the bottom billion” of population living in poverty by inducing disfiguration, loss of productivity, and a burden of 3.3 million disability-adjusted life years (3–5). More than 47 countries have reported cases of leishmaniasis with 0.2–0.4 million cases reported annually (6). The fatality rate for visceral leishmaniasis (VL) can become as high as 100% if left untreated (7). Several antileishmanial drugs, including amphotericin B, paromomycin, and miltefosine, against *Leishmania* are not fully effective, due to drug resistance, high toxicity, cost, and different modes of administration (8, 9). Therefore, vaccination remains an alternative for the prevention and control of this dreadful parasitic disease.

Several studies have demonstrated the critical role of a Th1 immune response that not only protects against the primary infection but also results in a lifelong immunity to re-infection (10, 11). CD4+ T-cells play a crucial role in immune protection by releasing a variety of cytokines, such as interleukin-12 (IL-12), gamma interferon (IFN-γ), tumor necrosis factor-α (TNF-α), and chemokines such as C–X–C motif chemokine ligand 10 (CXCL-10), and regulated on activation, normal T cell expressed and secreted (RANTES) (12). Although immune activation by CD8+ T-cells through granzyme activities and by the release of IFN-γ is reported, CD4+ T-cells are pre-dominantly involved in immune cell activation against *Leishmania* by producing IFN-γ, macrophage migration inhibitory factor, and tumor necrosis factor/lymphotoxin (TNF/LT) (13). Conversely, diverse cytokines such as interleukin-4, IL-10, and transforming growth factor β, have been shown to modulate Th1 responses, deter macrophage activation, and therefore exacerbate the disease (14, 15).

Many leishmanial targets have already been identified and vaccination with component proteins, such as CPs (16, 17), gp63 (18), LeIF (19), KmP-11 (20), ODC (21), and PDI (10), induces CD4+ T-cell-mediated cellular immune activation indicating a Th1-type immune response. Although many of these strategies have resulted in protection in the mouse model, with the exception of Leish-F1, many of them have either failed to prevent natural infection or have offered no protection in monkey and human models (22–25).

In spite of extensive information regarding various life stages of *Leishmania*, inter-specific diversity, host evasion mechanisms, and the heterogeneity of the population (26, 27), successful vaccine development against human VL remains an unprecedented challenge. Recent reports emphasize a subtle variation in antigen presentations as one of the plausible explanations for the failure of protective vaccines in human trials (28). This is the one area, especially the MHC mode of peptide presentation that has been mostly overlooked and might be the reason for the failure of most vaccine candidates (28). The current findings on the MHC-restricted epitope-based vaccines approach seem to be inducing more potent responses than whole protein vaccines (29, 30). Therefore, the reverse vaccinology approach remains elusive for the identification of specific human leukocyte antigen (HLA)-restricted epitopes.

The present study aimed to identify the potential immunogenic promiscuous CD4+ T cell epitopes by the reverse vaccinology approach. In this context, we hypothesized that a combination of modern sequence and protein structure algorithms would help the search for potential immunogenic epitopes that can induce CD4+ T-cell responses, which could be used to potentiate a human universal T-epitope vaccine against VL. As VL is the most serious form of *Leishmania*, we mined the exceedingly overexpressed protein in the amastigote condition of *Leishmania donovani* for the immunogenic CD4+ T-cell epitope. The antigenicity and immunogenic potential (IL-10 and IFN-γ) of peptides were further determined. The molecular docking approach was also utilized further to verify the antigen presentation. We identified the most important epitopes as P1-P6, which are capable of protecting against infection by *L. donovani* by evoking the cell-mediated immune response.

## Materials and Methods

### Selection of the *Leishmania* Antigens

The wide transcriptome analysis of *Leishmania* species revealed an alteration in their gene expression in the diverse stress conditions. Previously, we mined the genes derived from *L. donovani*, which are extensively overexpressed in the amastigote condition (31). For this study, six antigens, which are at least 10-fold upregulated, were retrieved from NCBI.^1^ The presence of N terminal signal peptides was evaluated by the SignalP 4.1 web server. The processed FASTA files (excluding signal peptide) were subjected to 15mer MHC class-II epitope prediction (HLA–DRB1*0101) by (I) SYFPEITHI (32), (II) IEDB (33), and (III) NETMHC-II 2.2 (34), according to the previously described methodology with certain modification (30). Primarily, high scoring peptides selected from each web server were included. The final set of consensus epitopes were selected for further study. To measure the sequence homology, BLASTP search was performed with a default parameter against human proteome (35, 36) and peptides with 80% query coverage and sequence identity, respectively, were excluded from the study.

### Prediction of Antigen Cross-Presentation and Theoretical Population Coverage Analysis

Human leukocyte antigen alleles are among the most polymorphic molecules in the human population. The efficiency of the immunogenic epitopes will be limited without HLA cross-presentation ability. Therefore, NetMHCpan3.4 (37) was used to evaluate the antigen cross-presentation. The theoretical population coverage of the selected epitope was calculated either individually or in a combination using the IEDB population coverage analysis tool.^2^ The consensus epitopes that are preferentially cross-presented by at least 98.5% theoretical population coverage were shortlisted for further analysis. As epitope conservancy plays a paramount role in the vaccine success, the conservancy of each shortlisted epitope was evaluated using the epitope conservancy tool^3^ available in the IEDB analysis resource.

### Peptide Structure Prediction and Docking Studies

Recently, a collective structure-sequence-based prophecy approach was pursued to improve the predictive ability (31, 38). The combination of these methods not only predicts the HLA binders but it also forecasts the docked epitope orientation. The optimal set of peptides (P1–P6) was modeled using the PEPFOLD web server (39). The endogenous 14 mer peptide complex with the crystal structure of HLA–DRB1 (PDB ID: 1AQD) was downloaded from the protein databank (PDB). The peptide was removed and used as a control peptide. The optimal set of peptides was then docked using the PatchDock web server (40) with HLA–DRB1 interacting residues as input. The antigenic properties of the optimal set of epitopes were evaluated by the Kolaskar and Tongaonkar Antigenicity method (41) accessible on the IEDB web server. It has already been reported that all T-cell and B-cell epitopes do not necessarily impair immunogenicity (42). Therefore, the ability of the shortlisted epitopes to modulate IFN-γ or IL-10 was predicted by the IFN epitope and IL-10Pred web server, respectively. The IFN epitope and IL-10Pred are *in silico* tools that can predict the nature of MHC class-II epitope as either an IFN-γ inducer or IL-10 inducer, respectively (43, 44).

### Molecular Dynamics Simulation

To study the intrinsic dynamics of atoms and molecules of the top ranked HLA-epitope complexes, molecular dynamics (MD) simulations were conducted using GROMACS v5.0 as described elsewhere (45, 46). A GROMOS 54A7 force-field was employed for amino acid interaction with the simulation of a water model by the simple point charge (SPC) method. Seven systems (including the control complex) were prepared by the addition of SPC water molecules (to cover the surface of each complex, 10 Å × 10 Å × 10 Å water box of cubic dimensions). To neutralize each system, a concentration of 0.15 M Na+/Cl− counter ions was added. Then the solvated systems were energy-minimized with 2,000 iterations on a convergence threshold of 1 kcal mol^−1^ using a steepest decent algorithm to remove unwanted steric clashes from all the complexes. After energy minimization, the systems were grouped into protein–peptide and solvent–ions to avoid collapse and then equilibrated for 1 ns in a constant volume (NVT). A constant temperature of 300 K was achieved using the Berendsen thermostat algorithm. After NVT, all systems were equilibrated for a further 1 ns at a constant pressure (NPT) of 1 bar using the Berendsen barostat. Long-range electrostatic interactions were computed using the particle mesh Ewald method with a cutoff of 1.2 nm, whereas short-range non-bonded interactions were calculated within a cutoff of 1.2 nm. For all systems, all bonds were constrained using the LINCS algorithm with an integration time-step of 2 fs. Finally, production MD simulations were performed using the NPT ensemble for 10 ns and the coordinates were saved every 2 ps for further analysis. To understand the dynamic stability of each system, the backbone root mean square deviation (RMSD) and radius of gyration (Rg) of the proteins were analyzed using the built-in utility tools of GROMACS. The gmx_hbond tool was employed to compute the total number of intermolecular H-bonds formed between HLA and epitopes during a 10-ns MD simulation.

### Principal Component Analysis

Principal component analysis (PCA) is a powerful tool in statistical mechanics employed to determine the correlated motions of the residues to a set of linearly uncorrelated variables called principal components (PCs). For the current study, the built-in utility tool gmx_covar of GROMACS was employed to calculate and diagonalize the covariance matrix for the coordinates of each complex whereas gmx_anaeig was used to analyze and plot the eigenvectors of each complex. All the 2-dimensional graphs generated were generated by the Grace V5.1.25 program.^4^

### Peptide Synthesis

The optimal set of epitopes was synthesized with more than 95% purity by Peptide2.0 (Chantilly, VA, USA). Lyophilized powder was dissolved in 10% dimethyl sulfoxide (Sigma-Aldrich, Germany) and stored aliquoted at −80°C until use.

### Soluble *Leishmania* Antigen (SLA)

Cultured *L. donovani* promastigote (200 × 10^6^ ml^−1^) in 5 ml of cold sterile phosphate-buffered saline (PBS) was subjected to five cycles of freeze and thaw in −195°C liquid nitrogen and 37°C water bath. Then, it was centrifuged at 10,000 × *g* for 20 min at 4°C [Afrin et al. (47)]. The supernatant containing SLAs were collected and protein concentration was measured by Lowry’s method and stored at −80°C until further use.

### Sample Collection and Peripheral Blood Mononuclear Cells Isolation

Six successfully cured VL subjects (treated with Amphotericin B) and six active VL subjects of both sexes aged 19–46 years were sampled from the outdoor patient department of Rajendra Memorial Research Institute. 10 ml of blood was collected each time in a sterile sodium EDTA vacutainer vial (BD Bioscience, CA, USA). The blood was stored at 4–8°C and processed within 4 h. The volunteers participated in this study donated blood more than once when required. Measurement of body temperature, body weight, total and differential WBC count, hemoglobin, blood sugar, serum creatinine, and prothrombin was performed in all cases. Each sample was diluted 1:1 with sterile PBS and loaded on Ficol-Hypaque 1077 (Sigma-Aldrich, Germany) and centrifuged at room temperature for 30 min at 400 × *g*. The separated mononuclear layer was washed twice with sterile PBS, counted, and utilized within 90 min. This study was carried out in accordance with the recommendations of the Institutional Human Ethical Committee of Rajendra Memorial Research Institute of Medical Sciences (RMRIMS) (Patna, India) with written informed consent provided by all participants. All subjects gave written informed consent in accordance with the Declaration of Helsinki. The protocol was approved by the Institutional Human Ethical Committee of the RMRIMS (Patna, India).

### Measuring the Intracellular Cytokines Produced from the CD4+ T-Cell against Shortlisted Epitopes

To monitor the intracellular cytokine produced from CD4+ T-cells in cured as well as from active VL subjects, PBMCs (1 × 10^6^ ml^−1^) were cultured in 12-well plates with either 20 µg/ml of individual peptides or with a cocktail of peptides overnight at 5% CO_2_. The challenge with SLA and a negative peptide was run simultaneously to all experiments as described previously (21). The cells were then challenged with a protein transport inhibitor, brefeldin-A (1 mg/ml), for 4 h. The cells were pelleted, washed with sterile PBS, and co-stained with anti-CD3-PerCP-conjugated antibodies (BD Biosciences) and anti-CD4-FITC (BD Biosciences) for 30 min at room temperature. The cells were then washed with stain buffer (PBS + 1% FCS) and fixed and permeabilized with Cytofix-Cytoperm buffer (BD Biosciences) for 20 min at 4°C. Afterward, the permeabilized cells were stained with either the anti-IFN-γ-PE or anti-IL-10-APC -conjugated antibody (BD Biosciences) for 30 min at 4°C and then washed with perm wash buffer (BD Biosciences). Same experimental concentration of appropriate isotype control was used to monitor the proper interpretation of results. The samples were processed through FACS-Calibur (BD Biosciences) and analyzed with CellQuest software. A logical gate set using a dot plot of side-scatter versus CD3 staining was used to evaluate CD3+ T-cells for the co-expression of CD4+ IFN-γ or IL-10. At least 30,000 cells were acquired for each analysis, and the results shown in percentage gated value (% gated).

### Enzyme-Linked Immunosorbent Assay (ELISA) for the Detection of the Anti-Peptide (P1–P6) Antibody in Active VL Subjects

The reactivity of peptides with active VL sera was measured by ELISA as described previously (48). Initially, three antigen concentrations (0.5, 1, and 1.5 µg per well) were used to examine the effective dose. Briefly, 96-well plates (Nunc) were coated with 1 µg/ml of each individual peptide in bicarbonate buffer (pH 9.6) and left overnight at 4°C. The coated wells were aspirated and blocked with 100 µl of 5% BSA solution for 2 h and washed three times with PBS-T. The presence of the anti-peptide antibody was evaluated by the serial dilution of active VL sera ranging from 1:25 to 1:51,000 (diluted twice each time). 100 µl of diluted sera was added to the respective well and incubated for 2 h at 37°C followed by three washings. The plates were aspirated and incubated for 1 h in HRP-tagged antihuman secondary antibody (1:5,000, in PBS-T) followed by the addition of 100 µl of the substrate (TMB) for 30 min. 50 µl of stop solution was added. The absorbance was measured at RT in the dark at 450 nm.

### Immunization Experiments

This study was carried out in accordance with the recommendations of the Animal Ethical Committee of RMRIMS, Patna, India. The protocols were approved by the institutional animal Ethical Committee of RMRIMS, Patna, India. Fifteen 5- to 7-week-old inbred male BALB/c mice were kept under sterile hygienic conditions in the animal house at RMRIMS. The mice were sampled (five per group) and immunized subcutaneously with either normal saline or peptide cocktail (50 µg) along with Freund’s complete adjuvant on Day 1. The peptide cocktail or SLA was dissolved in sterile saline (0.85% NaCl). The first booster and second booster immunizations were repeated with Freund’s incomplete adjuvant on Days 7 and 15, respectively. After 10 days of the second booster dose, the mice were sacrificed, the spleen cells were homogenized and mononuclear cells were isolated by Ficol-Hypaque 1077, as described previously.

### Measuring T-Cell Proliferation in the Experimental Animal Model

The T cell proliferation assay was carried out according to the protocol described earlier (38). Briefly, splenocytes (1 × 10^6^ per well) were isolated and stained with 2.5 mM CFSE (5-6-carboxyfluorescein diacetate succinimidyl ester, Biolegend) and cultured in RPMI-1640 complete medium. The stained cells were incubated at 37°C in 5% CO_2_ for 4 days in the presence or absence of *L. donovani* (1:10). Here, SLA was used as positive control. On Day 4, the cells were washed and stained with anti-CD3-PerCP. Samples were acquired on FACS-Calibur (BD Biosciences) and analyzed with CellQuest software, and a logical gate set using a dot plot of side-scatter versus CD3 staining was used to examine T cell proliferation.

### Intracellular Cytokines and Chemokines Produced from the CD4+ T-Cell in the Experimental Animal Model

The intracellular cytokines, such as IL-17, IL-22, IL-12, IFN-γ, and IL-10, were measured by the methodology described above with certain modifications. Briefly, the splenocytes were cultured in complete RPMI-1640 medium (Sigma-Aldrich) with appropriate antibiotics and *L. donovani* (1:30 ratio) for 48 h and with brefeldin-A (1 mg/ml) for the last 4 h. Anti-mouse monoclonal anti-CD3-PerCP and anti-CD4 conjugated with FITC were used as surface markers. Same experimental concentration of appropriate isotype control was used to monitor the proper interpretation of results. The cells were fixed and permeabilized with Cytofix-Cytoperm buffer (BD Biosciences) and stained with either PE conjugated IL-12, IFN-γ, or APC -conjugated IL-17, IL-22, and IL-10 for 30 min. The cells were washed and a logical gate set using a dot plot of side-scatter versus CD3 staining was used to measure the level of intracellular cytokine. At least 30,000 cells were acquired for each analysis and the results shown in percentage gated value (% gated).

### Effect of Vaccination on Antileishmanial Splenocytes Activity in Balb/c Mice

The splenocytes were seeded in 24-well plates (2 × 10^5^ cells per well) and challenged with *L. donovani* and cultured for 48 h. Changes observed in nitric oxide generation in the splenocytes of differentially vaccinated mice were measured by the Griess reagent as described previously (10). The amount of nitric oxide formed was measured in Molar/10^6^ (M/ml) cells and was calculated by comparing the standard sodium nitrite concentration curve. The lower limit of the sensitivity of the nitrite assay was 0.08 µM/ml.

### Statistical Analysis

All data were expressed as mean SD and the Student’s *t*-test was performed using the GraphPad prism software (*P*-values < 0.05 were considered significant) (31).

## Results

### *In Silico* Analysis of *L. donovani* Proteome

Based on the recently published annotation about the vaccine potential of amastigote specific overexpressed protein (31), six antigens that expressed at least 10-fold or more, were selected as candidate antigens (Table 1). Out of these, only one protein (XP_003863771) was found to contain N terminal signal peptide and the signal peptide was removed (49). The selected antigens were screened for plausible 15mer MHC class-II epitopes (HLA–DRB1*0101) using three different types of software. The threshold value for SYFPEITHI and IEDB was adopted from the published literature (50–52). Nonetheless, to avoid false positives, we only selected peptides with binding scores above 21 for SYFPEITHI and those with less than 3 for IEDB. Those peptides were further crosschecked with the NETMHC-II web server and the IC50 ≤ 50 nM (strong binding) was selected for further analysis. Based on the ranking scores, 37 consensus epitopes were shortlisted for further study (Table 2). One of the major obstacles in vaccine development is the sequence homology with humans. We further compared these predicted epitopes against *Homo sapiens* (taxid: 9606), to exclude such epitopes with the potential for generating autoimmune responses, but none of them had sequence identity and query coverage >80%, respectively.

**Table 1:** The candidate antigens were selected for the screening of MHC class-II-restricted epitopes.

Sl. no.	Protein-Id	Name	A:P ratio
1	XP_003865112.1	Hypothetical protein	11
2	XP_003862935.1	Hypothetical protein	50
3	XP_003863771.1	Hypothetical protein	13.6
4	XP_003858976.1	Phosphate-repressible phosphate permease-like protein	12.2
5	XP_003857910.1	Phosphoglycan beta 13 galactosyltransferase	11
6	XP_003858984.1	Protein kinase putative	15.8

**Table 2:** Characteristics of *in silico* predicted human leukocyte antigen–DRB1*0101-restricted 15 mer epitopes derived from shortlisted antigenic proteins of *Leishmania donovani*.

Sl. no.	Protein-Id	Peptide	Position	SYFPEITHI	IEDB	NETMHC-II
1	XP_003865112.1	QSKFRPISASSMPDE	231	34	1.15	SB
		IQSYRLLKDAVASPL	274	29	1.15	SB
		LQSFVVTCSAAALTL	431	25	0.19	SB
		QSFVVTCSAAALTLA	432	25	0.47	SB
		ALLQSFVVTCSAAAL	429	21	1.33	SB

2	XP_003862935.1	ILDVIFMTSRLVAKM	522	30	2.05	SB
		LRRLFSIRTNALARE	173	28	2.74	SB
		FDLFLFSNGAVVWWG	282	27	0.32	SB
		TLGFQPLAVEPALDR	353	26	0.71	SB
		NCFDLFLFSNGAVVW	280	25	1.06	SB
		RVRLRRLFSIRTNAL	170	24	0.77	SB

3	XP_003863771.1	LLALILLGGIGAVGY	563	36	1.33	SB
		YPVYPFLASNAALLN	312	33	0.14	SB
		LPSFHAMSAFHSAAK	399	32	0.6	SB
		NAALLNLIPSLLYRV	321	30	0.6	SB
		VYPFLASNAALLNLI	314	26	0.09	SB
		RALLALILLGGIGAV	561	25	2.05	SB
		AALLNLIPSLLYRVQ	322	22	0.6	SB

4	XP_003858976.1	LQVFTAICASFAHGA	332	33	2.27	SB
		FPFFSGVAPIVASWF	145	30	0.71	SB
		ERVFRYLQVFTAICA	326	28	2.74	SB
		KDDFPFFSGVAPIVA	142	27	2.18	SB
		DFPFFSGVAPIVASW	144	26	0.62	SB
		LESFFVLFKGASKRL	204	26	2.46	SB
		FFVLFKGASKRLKWS	207	25	2.09	SB
		FRYLQVFTAICASFA	329	24	2.09	SB
		SRGFSAELSAALVVS	411	24	1.06	SB
		RGFSAELSAALVVSF	412	24	1.36	SB

5	XP_003857910.1	LALLIMLYALIATQF	115	33	0.28	SB
		LIMLYALIATQFSDD	118	33	0.96	SB
		IMLYALIATQFSDDA	119	31	2.51	SB
		VSVLALLIMLYALIA	112	25	0.28	SB
		VLALLIMLYALIATQ	114	25	0.28	SB

6	XP_003858984.1	VSILRQLLSVTAHTH	383	32	0.77	SB
		KVAVSILRQLLSVTA	380	27	0.97	SB
		GISFSRAFAANIESA	127	26	2.82	SB
		YQFYHRARSYVIFTT	692	24	0.09	SB

*The consensus peptides with binding scores above 21 for SYFPEITHI less than 3 for IEDB and predicted IC50 ≤ 50 nM (Strong Binding) for NETMHC-II were selected for further analysis*.

### Selection of Potential Immunogenic Epitopes

Antigen cross-priming played a crucial role in the peptide-based therapeutic vaccine against *Leishmania* (53), and therefore, candidate epitopes were short listed with multiple HLA binding to increase the coverage of the target population. To measure the cross MHC class-II allele binding affinity of our selected epitopes, the NetMHC-II 2.2 web server was used, which is one of the best performing MHC-binding predictors. A total of 37 epitopes were selected with extensive binding affinity to the 26 diverse MHC class-II allele, including DRB10101, DRB10301, DRB10401, DRB10404, DRB10405, DRB10701, DRB10802, DRB10901, DRB11101, DRB11302, DRB11501, DRB30101, DRB40101, DRB50101, DPA10103-DPB10401, DPA10103-DPB10201, DPA10201-DPB10101, DPA10103-DPB10301_DPB10401, DPA10301-DPB10402, DPA10201-DPB105021, DQA10102-DQB10602, DQA10401-DQB10402,DQA10501-QB10201, DQA10501-DQB10301, DQA10301-DQB10302, and DQA10101-DQB10501 (Table S1 in Supplementary Material). Based on HLA cross-priming analysis, a total of six highly promiscuous epitopes, such as FDLFLFSNGAVVWWG (P1), YPVYPFLASNAALLN (P2), VYPFLASNAALLNLI (P3), LALLIMLYALIATQF (P4), LIMLYALIATQFSDD (P5), and IMLYALIATQFSDDA (P6), were selected which shared at least 98.5% of the worldwide population coverage (Table 3). We also evaluated the theoretical population coverage which was defined as the total number of selected peptide combinations against the worldwide population. Six potential immunogenic epitopes in combinations can be presented by 26 different MHC class-II alleles. Less than ~0.3% of the members of the average population have not shown any peptide binding. The immunogenic nature of the optimal set of promising epitopes was evaluated by IFN epitope and IL-10Pred. Interestingly, all the epitopes are found to be IFN-γ inducer, whereas most of the epitopes were IL-10 non-inducer (except P3).

**Table 3:** Theoretical population coverage analysis of individual peptide along with concerned proteins was analyzed.

Sl. No.	Protein-Id	Peptide	Population coverage (%)	Population coverage of the protein (%)
1	XP_003865112.1	QSKFRPISASSMPDE	66.53	98.71
		IQSYRLLKDAVASPL	81.94	
		LQSFVVTCSAAALTL	96.92	
		QSFVVTCSAAALTLA	96.24	
		ALLQSFVVTCSAAAL	98.08	

2	XP_003862935.1	ILDVIFMTSRLVAKM	97.43	99.60
		LRRLFSIRTNALARE	91.51	
		FDLFLFSNGAVVWWG	98.52	
		TLGFQPLAVEPALDR	89.04	
		NCFDLFLFSNGAVVW	97.91	
		RVRLRRLFSIRTNAL	83.33	

3	XP_003863771.1	LLALILLGGIGAVGY	59.45	99.17
		YPVYPFLASNAALLN	99.17	
		LPSFHAMSAFHSAAK	85.28	
		NAALLNLIPSLLYRV	98.44	
		VYPFLASNAALLNLI	99.00	
		RALLALILLGGIGAV	60.53	
		AALLNLIPSLLYRVQ	98.44	

4	XP_003858976.1	LQVFTAICASFAHGA	85.89	99.50
		FPFFSGVAPIVASWF	82.51	
		ERVFRYLQVFTAICA	97.60	
		KDDFPFFSGVAPIVA	94.43	
		DFPFFSGVAPIVASW	87.01	
		LESFFVLFKGASKRL	97.63	
		FFVLFKGASKRLKWS	87.08	
		FRYLQVFTAICASFA	98.39	
		SRGFSAELSAALVVS	93.41	
		RGFSAELSAALVVSF	90.98	

5	XP_003857910.1	LALLIMLYALIATQF	99.36	99.47
		LIMLYALIATQFSDD	98.69	
		IMLYALIATQFSDDA	98.87	
		VSVLALLIMLYALIA	83.98	
		VLALLIMLYALIATQ	93.53	

6	XP_003858984.1	VSILRQLLSVTAHTH	85.64	99.50
		KVAVSILRQLLSVTA	98.18	
		GISFSRAFAANIESA	93.46	
		YQFYHRARSYVIFTT	79.16	

**Figure 1:**
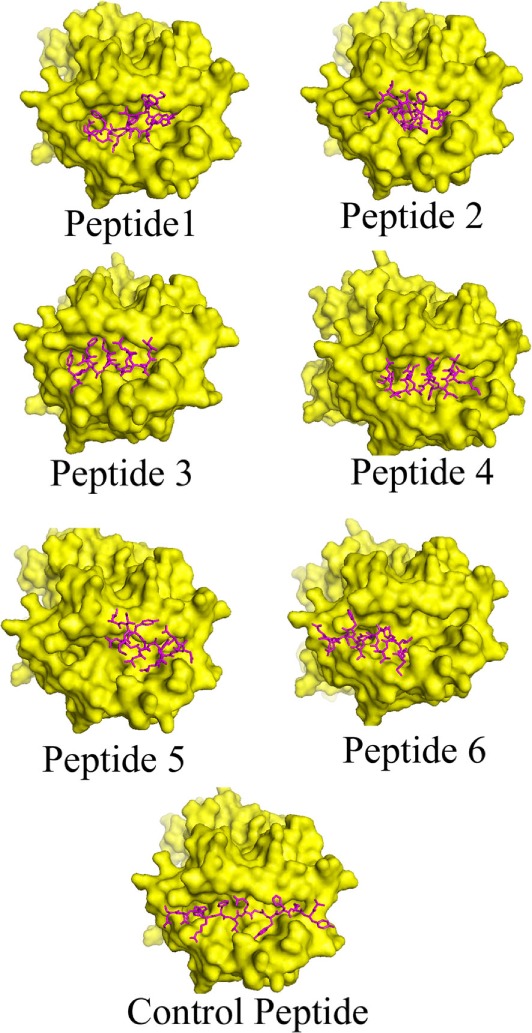
Binding modes of selected epitopes at the peptide-binding grooves of human leukocyte antigen (HLA)–DRB1*0101 molecules. Candidate peptides were predicted to locate onto the peptide-binding cleft of the HLA molecules by using fire dock web server. The structures of the HLA molecule is depicted in surface view mode where the peptides are represented in magenta color.

The interaction between the epitope and HLA is a typical characteristic and the availability of the crystal structures allows us to monitor the mode of interaction computationally (53). The data obtained from the PatchDock web server revealed that the anchor residues of most of the shortlisted epitopes interact with the active site residues of HLA–DRB1*0101, which indicate the preferential mode of presentation of the selected epitopes to the T cell receptor (Figure 1). The Geometric shape complementarity scores for P1, P2, P3, P4, P5, and P6 peptides were 9,044, 8,456, 8,142, 8,346, 8,120, and 7,796, respectively, which were very close to the score of the positive control peptide (10,932).

### Trajectory Analysis

**Figure 2:**
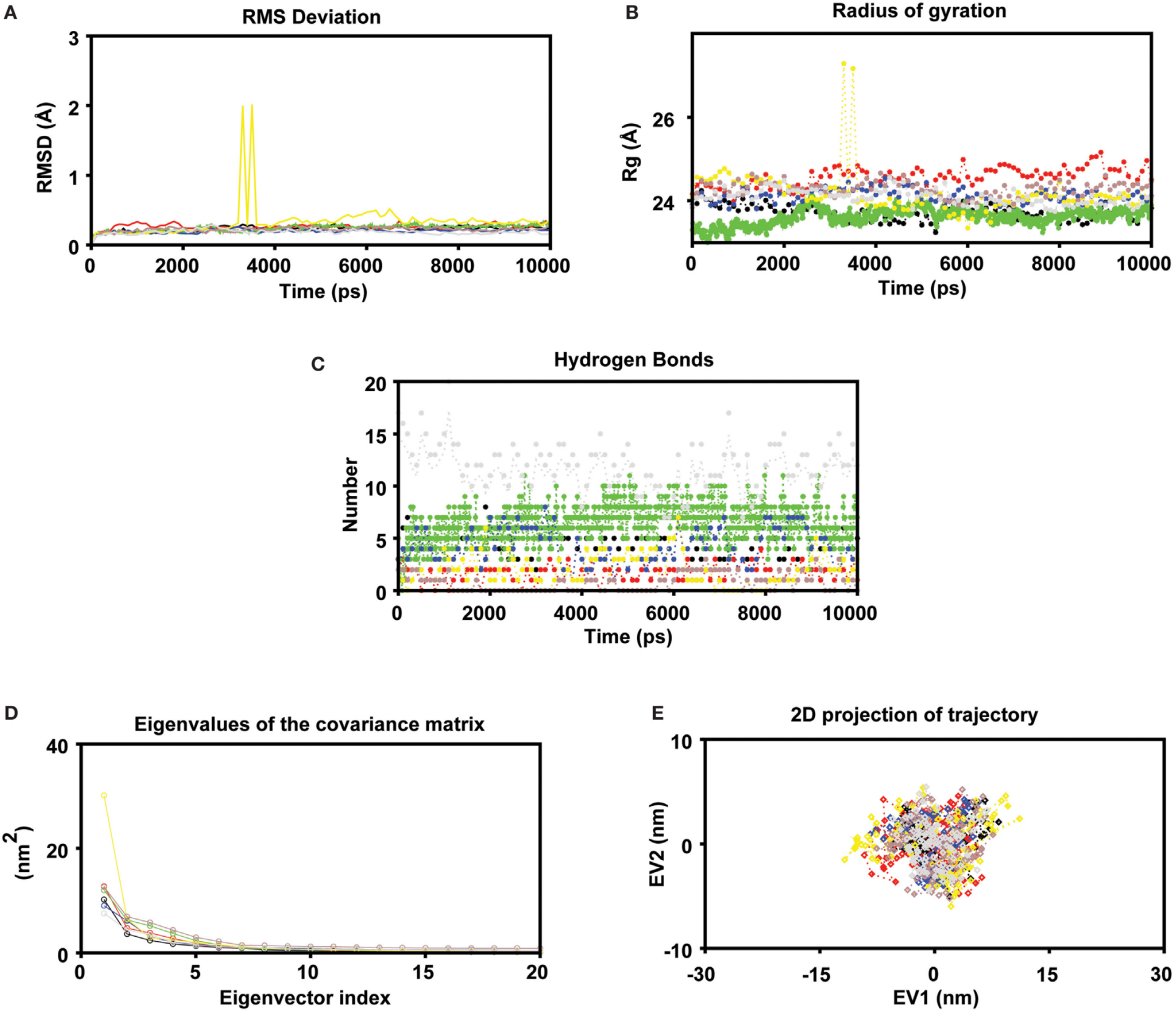
MD simulations were carried out in GROMACS v5.0 **(A)** Molecular dynamics simulation study of human leukocyte antigen (HLA)-epitope complexes displaying the root mean square deviation (RMSD) of the backbone atoms during 10 ns simulations. *Black, Red, Green, Blue, Yellow, Brown (epitope complex), and Gray (control) color represents RMSD of each HLA-Epitope complexes. **(B)** Radius of gyration of the protein as a function of the simulation time averaged over 10 ns MD **(C)** Number of intermolecular hydrogen bonds (H-bonds) formed between the epitope and HLA for the entire duration of simulation. The criteria to measure H-bonds are based on cutoffs for the hydrogen-donor–acceptor angle (30°) and the donor–acceptor distance (0.35 nm) (where OH and NH groups were regarded as donors; and O and N were acceptors) **(D)** Principal component analysis. The eigenvalues plotted against the corresponding eigenvector indices obtained from the Cα covariance matrix constructed from 10 ns of the MD trajectory. First 20 eigenvectors were used for calculations **(E)** Projection of the motion of the HLA-Epitope complexes in phase space along the first two principal eigenvectors (EV1 and EV2). The cloud represents the projection of trajectories eigenvectors (EV1 and EV2).

**Figure 3:**
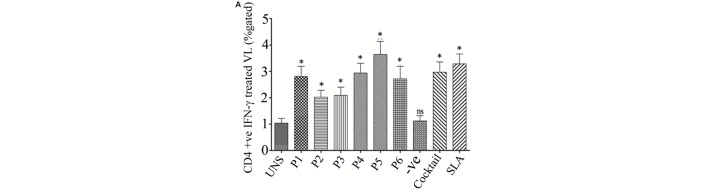

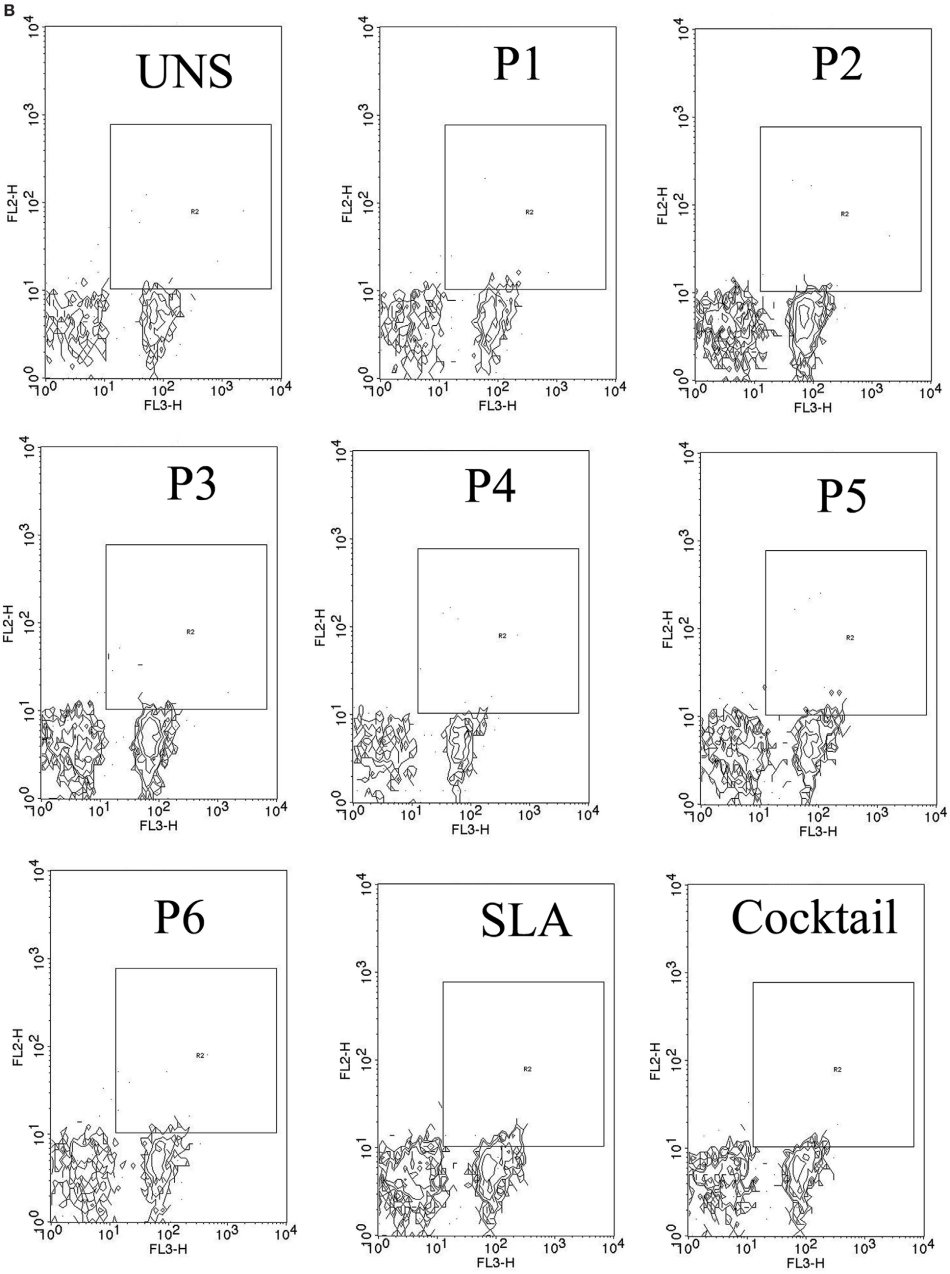
The ability of the optimal set of epitopes to modulate intracellular level of CD4+ IFN-γ was accessed by flow cytometry (% gated). PBMCs isolated from six treated visceral leishmaniasis (VL) subjects were cultured for overnight. The result suggested that the epitopes induces Th1 immune response against *Leishmania donovani* challenges. **(A)** Challenge with peptide either individually or as cocktail modulates the protective immune response by inducing CD4+ IFN-γ in successfully cured VL subjects. Here, soluble *Leishmania* antigen (SLA) was used as a positive control. **(B)** Representative FACS plot showing CD4+ T cell producing IFN-γ against the stimulation of CD4 T cell epitopes in treated VL subjects. Student’s *t*-test was used and *P*-value less than 0.05 was considered as significant value.

To measure the conformational stability and mode of epitope binding, a MD simulation of 10 ns was conducted for each complex. To access the dynamic stability of the systems, a RMSD profile for backbone residues were generated (Figure 2A). The backbone RMSD was found to be within 0.27 to ~1.98 Å during the simulation. The RMSD profile showed that all systems followed a stable deviation after 7 ns. A minute deviation was observed in the case of the P5 complex during the initial 3–4 ns, but the complex got stable after 7 ns at 0.43 Å until the end of the simulation. Further, it was found that, during the last 10 ns of the simulation period, on average all the systems showed a stable deviation with a RMSD about ~0.4 Å, indicating the stable nature of the complexes. The compactness of each system (properties linked to the molecular volume and compactness) was measured by plotting the Rg of the protein (as displayed in Figure 2B). Minute observation of the Rg plot depicts that most of systems had a compact trajectory with an average Rg of ~24.57 Å (Figure 2B). Like RMSD, the Rg also followed the same trend indicating the stable nature of the trajectories (with minute exceptions in the case of complex P5). The intermolecular hydrogen-bonds formed between protein and epitopes play a pivotal role in the stability of the protein–peptide systems. In most of the systems, the epitope binds to the central cavity of HLA groove, but they vary in terms of their molecular interactions through intermolecular hydrogen bonds (Figure 2C). For instance, the intermolecular hydrogen bonds formed in the control complex were on the higher side as compared to other complexes. More or less, in all complexes, the number intermolecular h-bonds were found to be in the increased trend during the second half of the simulation time: indicating stronger interactions at the active pocket (Figure 3). Although in some cases the numbers of H-bonds were not found to be stable, later they were well compensated by Van Der Waals and hydrophobic contacts. The number of H-bonds was found to be within ~2 to 10 with an average number of ~4.74.

### Essential Dynamics (ED)

To further support our MD simulation results, ED was performed to analyze the conformational dynamics (i.e., examine the overall motion of the protein molecules on each HLA-epitope system). As the first few eigenvectors capture the bulk of the internal motions, we retrieved the first two projections from the protein trajectories during 10 ns simulation and projected them onto the eigenvectors as obtained from the covariance matrices. Steep curves of eigenvalues (Figure 2D) were acquired after plotting the eigenvalues against the eigenvectors, where it was observed that 90% of the backbone motion was covered by the first 30 eigenvectors. To investigate the resultant MD trajectories, we performed ED and progressively added sequential time points to a scatter plot in the space of the two dominant PCs. The scatter plot generated for all the HLA complexes was found show non-significant variation among all the all systems studied (Figure 2E). These results indicate that the motions of the backbone reached their equilibrium fluctuations in the first 10 eigenvectors. The plot of the eigenvalues obtained from the diagonalization of the covariance matrix of the atomic fluctuations is displayed in Figure 2D. The first few eigenvalues (EV1 and EV2) are relative to concerted motions and quickly decrease in amplitude to reach a number of constrained and more localized fluctuations. The projection of the first two PCs displays the motion of the complex in the phase space (Figure 2E) where the overall flexibility is calculated by the trace of the diagonalized covariance matrix. The trace values of the covariance matrixes of the HLA–epitope complexes were found to be 29.87, 38.58, 29.13, 31.49, 37.19, 33.7 (complex), and 27.46 nm^2^ (control), respectively. The graph indicates the variance in the conformational distribution, where each dot represents one conformation of the complex. The unremitting color representation highlights the periodic jumps between these conformations.

### Epitope Conservancy Analysis

The epitope conservancy analysis revealed a moderate conservancy of these optimal set of epitopes across the different *Leishmania* species. More specifically, the epitope P1 was found to be the most conserved epitope, which shared 100% sequence identity with *L. infantum, L. major, L. mexicana*, and *L. brazilensis*. Interestingly, most of the epitopes (P4 shared 93.33% identity) are 100% conserved against *L. infantum*. Though the epitope conservancy varies from 53.33 to 100%, three epitopes (P1, P2, and P3) shared at least 80% conservancy against diverse *Leishmania* species (Table 4). Further analysis of the selected epitopes demonstrated a strong relative antigenicity. Precisely, P1, P2, and P3 shared high antigenicity whereas the last amino acid residue from P3 (phenylalanine) and two consecutive amino acid residues (i.e., phenylalanine and serine) fell in the non-antigenic region. Other than these two residues, the antigenicity score of each residue fell above the antigenic determinant threshold value of 1.0 (Figure S1 in Supplementary Material).

**Table 4:** The selected epitopes (P1–P6) conservancy analysis among diverse *Leishmania* species by IEDB conservancy tool.

Peptide name	*L. donovani*	*L. infantum*	*L. major*	*L. mexicana*	*L. brazilensis*
FDLFLFSNGAVVWWG (P1)	100%	100%	100%	100%	100%
YPVYPFLASNAALLN (P2)	100%	100%	93.33	80.00	86.67
VYPFLASNAALLNLI (P3)	100%	100%	93.33	86.67	93.33
LALLIMLYALIATQF (P4)	100%	93.33	66.67	80.00	73.33
LIMLYALIATQFSDD (P5)	100%	100%	66.67	80.00	53.33
IMLYALIATQFSDDA (P6)	100%	100%	66.67	80.00	53.33

*Here, P1 was found to be highly conserved among all the selected species. All the selected epitopes (except P4) shared 100% identity with L. infantum*.

### Validation of Synthetic Multi-Epitope Peptides Immunogenicity in VL Subjects

**Figure 4:**
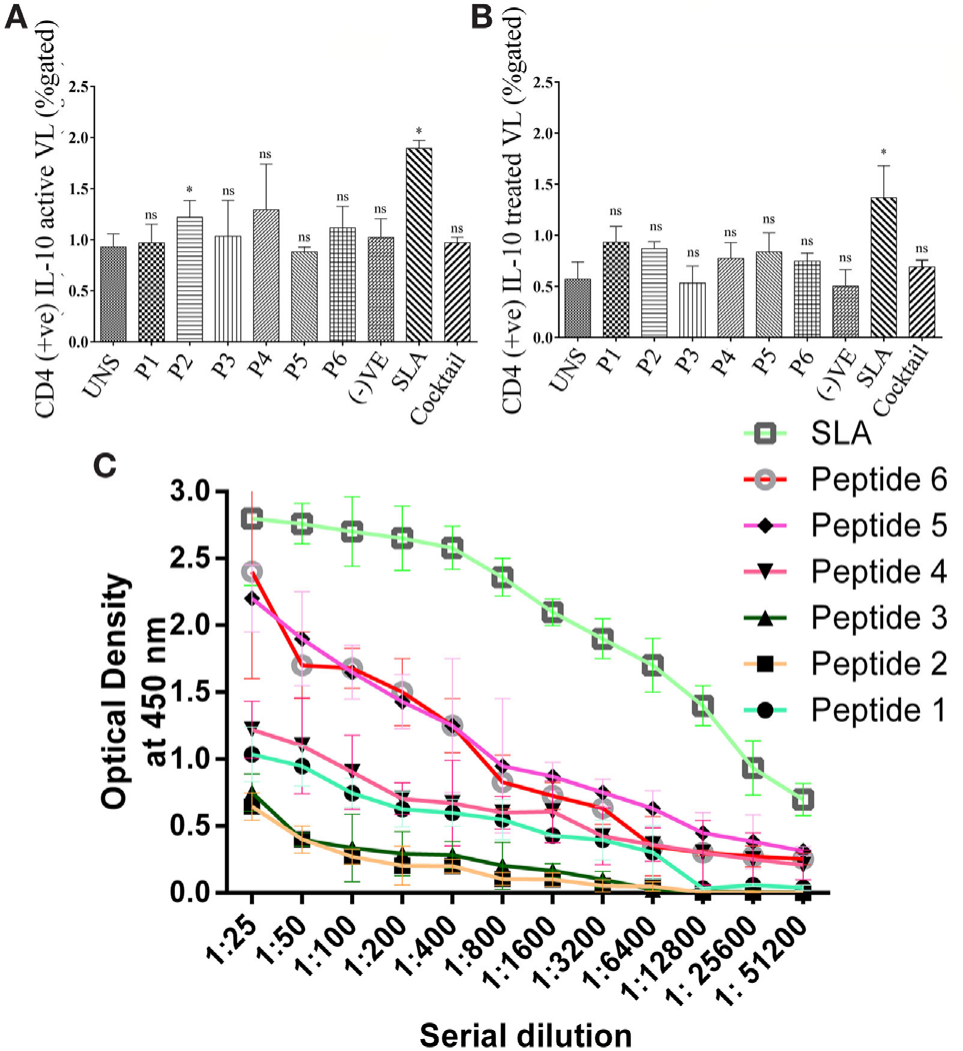
The intracellular level of CD4+ IL-10 was measured in active visceral leishmaniasis (VL) subjects (*n* = 6) as well as in treated VL subjects (*n* = 6). Here, the PBMCs were used to stimulate IL-10 production. **(A)** All the peptides except P2 did not induce IL-10 in active VL subjects. **(B)** However, none of the peptide have immuno-pathogenic role by modulating the intracellular level of IL-10 in treated VL subjects. In both active and treated VL subjects, no impact of cocktail of peptide was found to modulate this Th2 cytokine. **(C)** Specific IgG class of antibodies was measured in the serum of active VL cases against optimal set of epitopes in order to evaluate peptide effect on humoral immune response. Specifically, P1, P4, P5, and P6 were found to have high antibody titer which gradually decreases with serial dilution. In particular, the epitopes, P5 and P6 derived from phosphoglycan beta 13 galactosyltransferase revealed a high OD value at 1:25 dilution which was very close to the positive control. *P*-value less than 0.05 was considered as significant value.

To validate the intracellular cytokine levels of IFN-γ and IL-10, following stimulation with six predicted immunogenic epitopes, the cultured PBMCs were analyzed. The results obtained from the FACS analysis (expressed in % gated cell) revealed that the stimulation of PBMCS with these peptides significantly triggered CD3+ CD4+ cells for intracellular IFN-γ production: indicating a Th cell driven toward the Th1 type. No such increased IFN-γ production was observed either in the unstimulated (UNS) culture condition or in the negative control (Figures 3A,B). In contrast to IFN-γ production, very low levels of intracellular IL-10 were detected *in vitro* with each synthetic peptide (except P2) against treated VL as well as in fresh VL subjects (Figures 4A,B). However, PBMCs isolated from fresh VL subjects who were stimulated with P2 peptide responded with a significantly upregulated IL-10 production. Conversely, the scenario was quite adverse in treated VL subjects (Figure 4B). No significant change in IL-10 production against P2 was observed in treated VL subjects. Though almost all the peptides react with active VL sera, P1, P4, P5, and P6 were found to have a high antibody titer, which gradually decreases with serial dilution. In particular, the epitopes, P5 and P6 derived from Phosphoglycan beta 13 galactosyltransferase, revealed a high OD value at 1:25 dilution, which was very close to the positive control. In contrast, P2 and P3 weakly induced the production of IgG in active VL cases (Figure 4C).

### Measuring the Immuno-Dominance of a Cocktail of Epitopes in Immunized Mice

**Figure 5:**
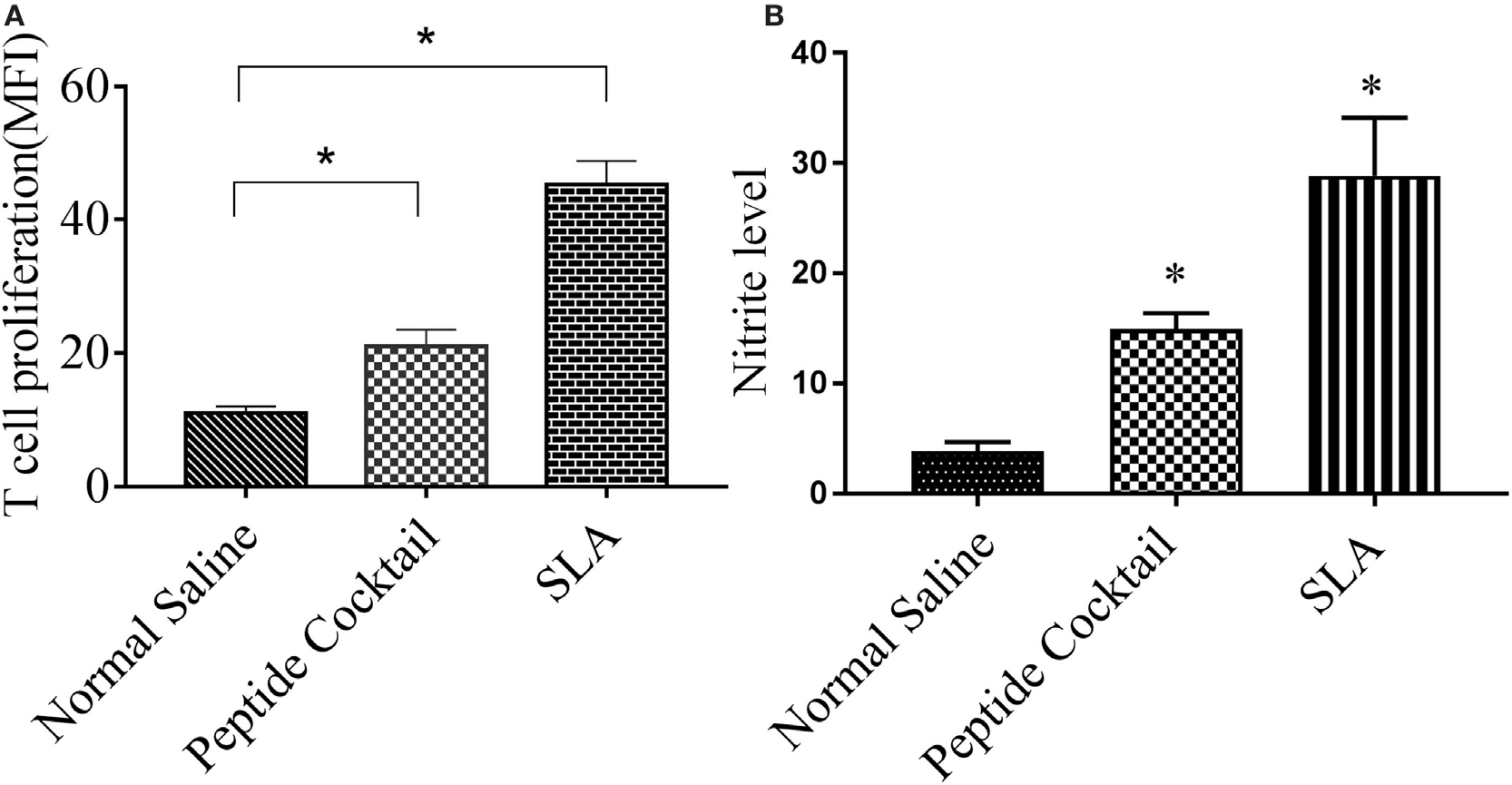
The efficacy of the cocktail of peptide to trigger the proliferation of T cell in immunized mice was measured by FACS. Briefly, the splenocytes were stained with CFSE, cultured for 4 days with *Leishmania donovani*, and co-stained with anti-mouse anti-CD3 PerCp. **(A)** A significantly enhanced proliferated T lymphocytes was observed in response to cocktail of epitopes stimulations with respect to normal saline. Also, an enhanced T cell proliferation was observed in soluble *Leishmania* antigen (SLA)-immunized splenocytes (positive control). **(B)** Effect of peptide immunization on antileishmanial splenocytes function was measured by the level of nitrite production in different vaccinated group. Here, an enhanced nitrite level in peptide vaccinated group was observed as compared to normal saline vaccinated group. *P*-value less than 0.05 was considered as significant value.

**Figure 6:**
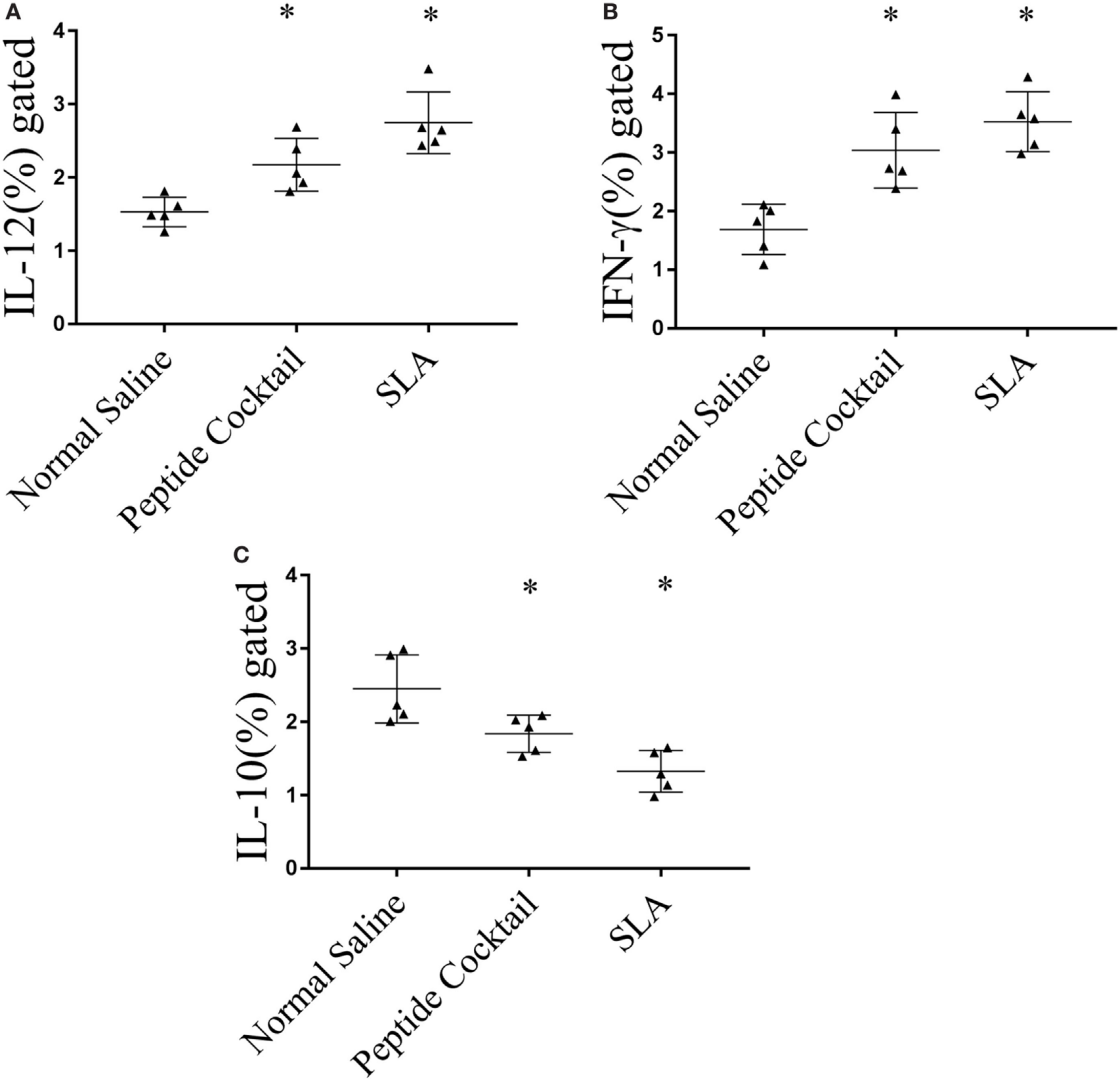
Cytokine immune responses generated in immunized mice were measured by FACS. Splenocytes derived from epitopes vaccinated mice explicitly **(A)** interleukin-12 (IL-12) and **(B)** IFN-γ as compared to normal saline vaccinated group. The soluble *Leishmania* antigen (SLA)-vaccinated group was also showed a significantly enhanced IL-12 as well as IFN-γ production as compared to normal saline vaccinated group. **(C)** In contrast, a significant reduction in IL-10 level was observed in epitope as well as in SLA vaccinated group. *P*-value less than 0.05 was considered as significant value.

**Figure 7:**
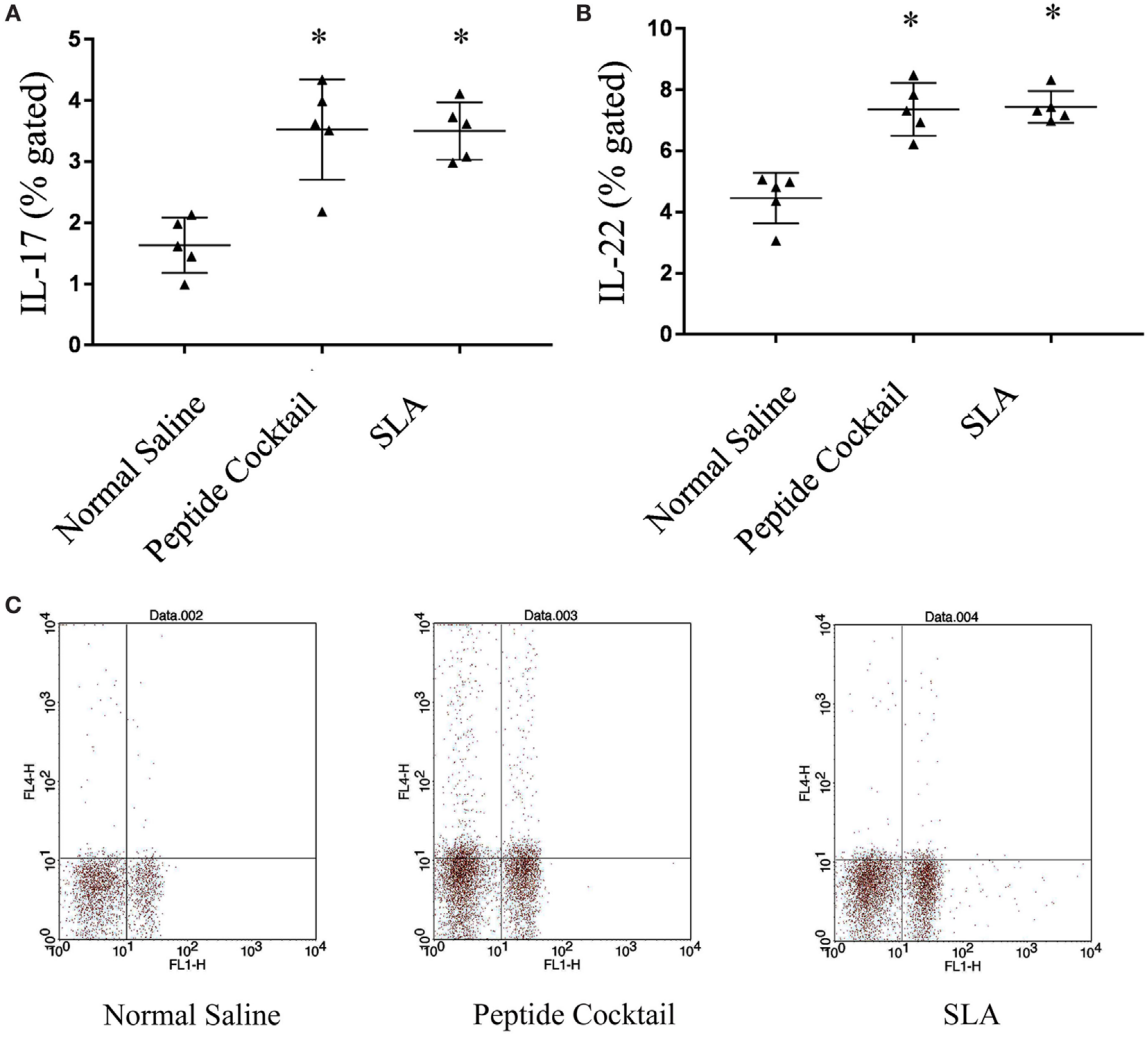
The effect of cocktail of peptide immunization on IL-17 and IL-22 was observed in the different immunized groups of mice. **(A,B)** The stimulation with *Leishmania donovani* induces a strong IL-17 and IL-22 level in cocktail of peptide immunized group as compared to normal saline immunized group. The soluble *Leishmania* antigen (SLA)-vaccinated group was also showed a significantly enhanced IL-17 as well as IL-22 production as compared to normal saline vaccinated group. **(C)** Representative FACS plot showing CD4+ T cell producing IL-17 against the stimulation of *L. donovani* in different vaccinated group. *P*-value less than 0.05 was considered as significant value.

Since the availability of blood samples from treated VL subjects is limited to broad-scale analysis of immuno-dominance and the immunogenicity of the peptide, experiments were carried out in the BALB/c mice model by immunizing a cocktail of epitopes with CFA/IFA. Ten days after the second booster dose, T cell proliferation was assessed. As shown in Figure 5A, splenocytes derived from epitope-vaccinated mice explicitly proliferated at a high responder rate compared to those from the normal saline vaccinated group. The SLA-vaccinated group also showed a significantly enhanced T cell proliferation compared to the normal saline vaccinated group. Thus, the results indicate the proliferative nature of the candidate peptides that could effectively induce spleen cell proliferation. Since the designed epitopes evoke an immune-dominant proliferative T-cell response, we further investigated whether the optimal set of epitopes was coupled with measurable cytokine production in *ex vivo* re-stimulated splenocytes cultures. Such cytokine production would aid in the development of functional epitopes-based assays to evaluate the type and maintenance of CD4+ T-cell populations in various leishmaniasis immunization models. Accordingly, splenocytes from immunized and infected BALB/c mice were assayed for the induction of epitope-specific Th1, Th2, or Th17-type cytokine responses by a FACS analysis. As shown in Figure 6, PBMCs from immunized mice induced an elevated level of CD4+ IL-12 and IFN-γ upon challenge with *L. donovani* (Figures 6A,B). In addition, the same group induced a high level of IL-17 and IL-22 (Figures 7A-C). However, the scenario was adverse in the case of IL-10: a Th2 cytokine. A significant reduction in the IL-10 level was observed in the cocktail of epitopes as well as in the SLA vaccinated group (Figure 6C). These findings suggest that the cocktail of epitopes-immunized mice induces a combined Th1/Th17-type CD4+ T-cell response. The synergistic role of IL-17A and IFN-γ to potentiate NO production and leishmanicidal activity in infected macrophages has already been established (54). To determine whether the synergistic role of IL-17A and IFN-γ could induce NO production, the level of nitrite was measured in the supernatants of parasite-challenged splenocytes isolated from mice in different vaccinated groups. As expected, significant nitrite production was observed in the cocktail of epitope-immunized mice, whereas in the normal saline immunized group, the production of nitrite was weakly induced (Figure 5B). All these results indicated the protective nature of the selected epitopes against VL.

## Discussion

So far, various techniques have been applied to address the need for antileishmania vaccine candidates that are effective against leishmaniasis. Most of the vaccine candidates became unsuccessful due to various factors such as poor antigen response, a lack of good animal models, and a lack of standardization (55). An excellent vaccine should also able to generate a promiscuous response against diverse *Leishmania* species, but normally only one species has been targeted at a time. Although using the whole parasite as a vaccine showed some potential as being safe and effective, it is afflicted by the issue of dose standardization. Thus, implementation of the immunome-derived vaccine approach, conjoined with *in vitro*/*ex vivo* validation may speed up the development of candidate vaccines for leishmaniasis (56–60). Only a few studies have been conducted to screen out CD4 T-cell epitopes from *Leishmania* spp. proteomes (61–63). However, most of these studies have focused on both CD4+ and CD8+ T-cell responses rather than only on CD4+ T-cells. This study was mainly intended to discover potentially immunogenic CD4+ T-cell epitopes derived from the essential genes of *L. donovani*.

Many overexpressed genes were recently analyzed by our group and we also screened the potentiality of the epitopes derived from hypothetical proteins as vaccine candidates in the context of the MHC class I allele (31). The findings obtained from the proteome analysis of six antigens revealed a set of 37 potential HLA–DRB1*0101 restricted immunogenic peptides which are in agreement and are coupled with Trost et al. theory (64). Notably, among the 10% top ranked predicted consensus epitopes identified by SYFPEITHI and BIMAS, a sizable proportion as high as 85% have the ability to activate the immune cells (65). Our previous study has also re-affirmed the immunogenicity of the consensus epitopes directed against VL (31, 38, 52). Therefore, it can be speculated that a major bulk of epitopes can be instrumental in generating the desired immune response. The only concern is that evaluation of its immunogenicity *in vitro* is not so easy. This necessitated the employment of a comprehensive immunoinformatics approach in the present study to identify a few potential hotspots in the form of shortlisted epitopes, which can be experimentally tested for their immunogenicity. To start up, BLASTP analysis was employed and the plausible cross-reaction of selected epitopes with human antigens was minimized. Notably, none of these was obscured to be 100% homologs with human antigens. Due to the high polymorphic nature of HLA (66), we focused on the antigen cross-priming, as it played a crucial role in a peptide-based therapeutic vaccine against *Leishmania* (53). There were many reasons for selecting the DRB1 gene for this study. First, DRB1 genes are normally expressed fivefold more than that of DRB3, DRB4, or DRB5 genes and they can frequently be cross-presented by other DR allele (67). We used NetMHC-II 2.2 in the present study to assess the cross HLA-binding affinity. Silent observations obtained in this regard revealed that, specifically, the epitopes P1, P2, and P3 derived from different hypothetical proteins had the ability to be cross-presented by at least 17, 22, and 19 HLA allele, respectively. In addition, we observed that peptides, such as P4, P5, and P6, derived from phosphoglycan beta 1,3 galactosyltransferase preferentially interacted with at least 18 and 21 HLA allele each, respectively. Concomitantly, the IC50 values of the shortlisted epitopes were below 500 nM, and we presumed that the larger peptide specificities among HLA allelic families might trigger desired immune responses in a comparatively large portion of a given population. This prompted us to screen the effects of the cocktail and we were able to demonstrate that the combination of these optimal set of epitopes shared ~99.7% theoretical population coverage.

Molecular mechanics methods such as molecular dynamics simulation and principal component analysis were employed to understand the dynamics and mode of epitope binding in HLA grooves. Further, the predicted RMSD and Rg of the complexes displayed a least deviation indicating the stable nature of the complexes, which was well supported by PCA analysis. In conclusion, with the help of different quantum mechanics approaches, it was observed that no major structural changes occurred upon epitope binding to HLA: indicating the structural stability of these complexes. Therefore, it can be concluded that the selected peptides will be preferentially presented to the TCR, which may evoke the cellular immune response. The Th1 type of immune response and its cytokines, such as IL-2, IL-12, IFN-γ, and TNF-α, are instrumental in generating protective immunogenicity against *L. donovani* (68). Hence, the ability of the predicted epitopes to induce Th1/Th2 cytokines (IFN-γ/IL-10) was examined by *in silico* tools. Striking revelations made from computational analysis revealed that most of the peptides were pre-dominantly IFN-γ inducer and IL-10 non-inducer peptides. The same was not true for the peptide P3, which was an IL-10 inducer peptide. Despite the IL-10 inducing property of P3, the same peptide was also found to be an IFN-γ inducer against other cytokines. It is well known that the fate of the disease mainly relies on the Th1 versus Th2 cytokine balance (69, 70) and the clinical cure is deeply associated with sustained elevated IFN-γ and downregulated IL-10 levels (70). All of this information suggests that these optimal set of epitopes with the exception of P3 are possibly highly immunogenic with the added advantages of exhibiting a HLA cross-presentation ability. Thus, these peptides may be a strong choice for a polytope-based vaccine candidate against VL.

Furthermore, the antigenic propensity analysis revealed that the antigenicity of most of the residues of the selected epitopes was above the threshold level of 1.0. Although Phenylalanine from P4 and two consecutive amino acid residues, i.e., phenylalanine and serine from P5 and P6, fell in the non-antigenic region, the antigenicity score was very near to the threshold value (0.975 each). This is of considerable importance since the estimation of such epitopes conserved among the diverse *Leishmania* species could possibly enhance its applicability. Given the low phylogenetic diversity between other visceral causing *Leishmania* species like *L. infantum*, it is possible that the same set of epitopes could elicit a common pattern of protective immune response (31). A support for this notion came from epitope conservancy analysis, which showed that all the epitopes (except P4) shared 100% sequence homology with *L. infantum* (Table 4). Interestingly, the epitope P1 was the most conserved among *L. donovani, L. infantum, L. major, L. mexicana*, and *L. brazilensis*. Apart from P1, the two other epitopes (P2 and P3) were also located in the conserved segments across the *Leishmania* species (Conservancy ≥ 80%). Therefore, the results obtained from this study suggest that the use of these conserved epitopes may offer a broader protection against *Leishmania* parasites.

To assess the probable effect of epitope-specific memory CD4+ T-cells in protection, PBMCs of treated VL subjects were stimulated with epitopes. In agreement with IFN epitope predictions, CD4+ T-cells are augmented with an elevated level of IFN-γ against both individual peptides and a cocktail of these peptides. Therefore, it can be postulated that these MHC class-II-specific epitopes are preferentially presented to the TCR–CD3 complex on CD4+ T-cells during the earlier course of VL infection. Such encouraging observations recommend the significance of the optimal set of epitopes as a Th1 cytokine modulator. This was well-reflected by their inability to modulate IL-10 in treated VL subjects. Against fresh VL, the scenario was also quite similar (except P2). Therefore, in consideration of the effects observed for these peptides individually on CD4+ T-cell activation in patients who had already cleared the *Leishmania* infection and the significance earlier shown for CD4+ T-cells and also in the present study, one can cogitate about the protective role of the MHC class-II-specific peptides identified during this study against VL. Moreover, a specific IgG class of antibodies was detected in the serum of active VL cases in order to evaluate the peptide effect on humoral response. The active VL subjects, that are positive to recombinant K39 (rK39) indicate that the anti-peptide antibodies have already been generated. The presence of the IgG antibody against the selected epitope will add a collegial effect in host protection.

Furthermore, we worked with a vaccination protocol and again observed that stimulation with the cocktail of epitopes against vaccinated mice resulted in a high degree of proliferating T cells. This signifies that a high percentage of the memory repertoire eventually participated in the response. This higher sensitivity in T cell proliferation might lead to an increased production of factors (IL-12 and IFN-γ) that are involved in the protective immune response. These data were further supported by an extensively elevated IL-12 produced in the epitope-vaccinated group. As already known, IL-12 is acting as a strong Th1-inducing adjuvant and plays a decisive role in Th1 differentiation (71). In fact, it was observed that splenocytes of the vaccinated hosts exhibited a significantly increased IFN-γ production by CD4+ T-cells. Therefore, it can be contemplated that the synergistic role of IL-12 was attributed to an increased accumulation/production of IFN-γ. In addition, the increased levels of IL-17 and IL-22 producing CD4+ T-cells against the epitope-vaccinated group signify the ability of these epitopes to modulate co-stimulatory molecules. Although controversial, in other forms of leishmaniasis, IL-17 produced by Th17 cells is shown to exacerbate the disease: suggesting a role in pathogenesis (72). Despite the findings, in recent years, the double knockout Il17ra−/− mice model is more susceptible to pathogenesis by down-modulating IFN-γ-expressing CD4+ T-cell frequencies (54). In line with this finding, other reports also claimed the complementary roles of IL-17 and IL-22 with Th1 cytokines in defense against VL (73). Therefore, it can be speculated whether the increased IL-17, IL-22, and IFN-γ levels by the CD4+ T-cells can have a synergistic protective effect in the down modulation of the intracellular IL-10. Furthermore, the supernatant of parasite stimulated splenocytes induces nitrite production, indicating a possible activated state of splenic macrophages in the groups vaccinated with the cocktail of epitopes. The low production of nitrite in the normal saline vaccinated group implied that, due to the absence of antigenic fragments during immunization, the macrophages failed to be properly activated. Thus, the result obtained from this study strengthen the notion that the apparent pro-inflammatory (Th1) cytokine induced NO production against the optimal set of epitopes may control the parasite replication (74). Altogether, our investigation yielded some protective CD4 restricted epitopes that produce an elevated amount of IL-12, IL-17, IL-22, IFN-γ/IL-10 ratio, and NO production. These MHC class-II-restricted epitope hotspots of *Leishmania* antigens with an ability to re-strengthen macrophage function through reverting impaired T cell proliferation and modulating preferential higher expansion of Th1 and Th17 CD4+ T-cell populations are promising for the design of a novel polytope vaccine for the control of VL. However, a detailed investigation of the epitopes in humanized mouse models is needed to explore their ability as a polytope-based vaccine candidate.

## Ethics Statement

This study was carried out in accordance with the recommendations of Institutional Human Ethical Committee of the Rajendra Memorial Research Institute of Medical Sciences (Patna, India) with written informed consent from all subjects. All subjects gave written informed consent in accordance with the Declaration of Helsinki. The protocol was approved by the Institutional Human Ethical Committee of the Rajendra Memorial Research Institute of Medical Sciences (Patna, India). This study was carried out in accordance with the recommendations of Animal Ethical Committee of Rajendra Memorial Research Institute of Medical Sciences (RMRIMS), Patna, India. The protocols were approved by the institutional animal Ethical Committee of Rajendra Memorial Research Institute of Medical Sciences (RMRIMS), Patna, India.

## Author Contributions

Conceived and designed the experiments: PD, SB, SD, and MD. Performed the experiments: MD, AK, FJ, and AR. Analyzed the data: MD, BD, SB, and PD. Contributed reagents/materials/analysis tools: PD, RT, KP, VD, and GS. Wrote the paper: MD, SB, and SD.

## Conflict of Interest Statement

The authors declare that the research was conducted in the absence of any commercial or financial relationships that could be construed as a potential conflict of interest.

## Footnotes

^1^http://www.ncbi.nlm.nih.gov/.

^2^http://tools.iedb.org/tools/population/iedb_input.

^3^http://tools.immuneepitope.org/tools/conservancy/iedb_input.

^4^http://plasma-gate.weizmann.ac.il/Grace/.

## Supplementary Material

Figure S1 The predicted antigenicity of the optimal set of epitopes by Kolaskar and Tongaonkar Antigenicity method available at IEDB web server. The yellow color represents antigenic regions.
