# Supplementary material for: Identification of Potential MHC Class-II-Restricted Epitopes Derived from Leishmania donovani Antigens by Reverse Vaccinology and Evaluation of Their CD4+ T-Cell Responsiveness against Visceral Leishmaniasis
Source: Front Immunol. 2017 Dec 14;8:1763. doi: 10.3389/fimmu.2017.01763 (PMC5735068; doi:10.3389/fimmu.2017.01763)
Supplement: Supplementary file 1 [file table_1.pdf]

TableS1: Analysis of cross HLA binding of the selected peptides.

Sl. No	Protein-Id	Peptide	HLA Cross-presentation	No. of cross binding HLA allele
**1**	XP_003865112 .1	QSKFRPISASSMPDE	HLA-DRB10101,HLA-DRB10401,HLA-DRB10404,HLA-DRB10405,HLA-DRB10701,HLA-DRB10802,HLA-DRB10901,HLA-DRB11101,HLA-DRB30101,HLA-DRB50101, HLA-DQA10501-DQB10301	11
		IQSYRLLKDAVASPL	HLA-DRB10101,HLA-DRB10301,HLA-DRB10401,HLA-DRB10404,HLA-DRB10405,HLA-DRB10701,HLA-DRB10802,HLA-DRB10901,HLA-DRB11101,HLA-DRB11302,HLA-DRB11501,HLA-DRB40101,HLA-DRB50101,HLA-DPA10201-DPB10101,HLA-DPA10301-DPB10402, HLA-DQA10501-DQB10301	16
		LQSFVVTCSAAALTL	HLA-DRB10101,HLA-DRB10401,HLA-DRB10404,HLA-DRB10405,HLA-DRB10701,HLA-DRB10802,HLA-DRB10901,HLA-DRB11101,HLA-DRB11302,HLA-DRB11501,HLA-DRB30101,HLA-DRB50101,HLA-DPA10103-DPB10401,HLA-DPA10103-DPB10201,HLA-DPA10301-DPB10402,HLA-DQA10102-DQB10602,HLA-DQA10501-DQB10301	17
		QSFVVTCSAAALTLA	HLA-DRB10101,HLA-DRB10401,HLA-DRB10404,HLA-DRB10405,HLA-DRB10701,HLA-DRB10802,HLA-DRB10901,HLA-DRB11101,HLA-DRB11302,HLA-DRB11501,HLA-DRB50101,HLA-DPA10103-DPB10401, HLA-DPA10103-DPB10201,HLA-DQA10102-DQB10602,HLA-DQA10501-DQB10301	15
		ALLQSFVVTCSAAAL	HLA-DRB10101,HLA-DRB10401,HLA-DRB10404,HLA-DRB10405,HLA-DRB10701,HLA-DRB10802,HLA-DRB10901,HLA-DRB11101,HLA-DRB11302,HLA-DRB11501,HLA-DRB30101, HLA-DPA10103-DPB10401,HLA-DPA10103-DPB10201,HLA-DPA10301-DPB10402,HLA-DQA10102-DQB10602,HLA-DQA10501-DQB10301	16
**2**	XP_003862935 .1	ILDVIFMTSRLVAKM	HLA-DRB10101,HLA-DRB10301,HLA-DRB11302,HLA-DRB10401,HLA-DRB10404,HLA-DRB10701,HLA-DRB11101, HLA-DRB11501,HLA-DRB50101,HLA-DPA10103-DPB10401,HLA-DPA10103-DPB10201,HLA-DPA10201-DPB10101,HLA-DPA10103-HLA-DPB10301_DPB10401,HLA-DPA10301-DPB10402,HLA-DQA10102-DQB10602,HLA-DQA10501-DQB10301	16
		LRRLFSIRTNALARE	HLA-DRB10101,HLA-DRB10301,HLA-DRB10401,HLA-DRB10404,HLA-DRB10405,HLA-DRB10701,HLA-DRB10802,HLA-DRB10901,HLA-DRB11101,HLA-DRB11302,HLA-DRB11501,HLA-DRB40101,HLA-DRB50101,HLA-DPA10201-DPB10101 ,HLA-DPA10301-DPB10402,HLA-DQA10102-DQB10602	16
		FDLFLFSNGAVVWWG	HLA-DRB10101,HLA-DRB10401,HLA-DRB10404,HLA-DRB10405,HLA-DRB10701,HLA-DRB10901,HLA-DRB11101,HLA-DRB11302,HLA-DRB11501,HLA-DRB30101,HLA-DRB50101,HLA-DPA10103-DPB10201, HLA-DPA10201-DPB10101,HLA-DPA10103-HLA-DPB10301_DPB10401,HLA-DPA10301-DPB10402 , HLA-DQA10102-DQB10602,HLA-DQA10501-DQB10301	17
		TLGFQPLAVEPALDR	HLA-DRB10101,HLA-DRB10401,HLA-DRB10404,HLA-DRB10405,HLA-DRB10802,HLA-DRB10901,HLA-DRB40101,HLA-DRB50101, HLA-DPA10201-DPB10101,HLA-DQA10102-DQB10602,HLA-DQA10301-DQB10302,HLA-DQA10401-DQB10402,HLA-DQA10501-DQB10201,HLA-DQA10501-DQB10301	14
		NCFDLFLFSNGAVVW	HLA-DRB10101,HLA-DRB10401,HLA-DRB10404 HLA-DRB10405,HLA-DRB10701,HLA-DRB10901,HLA-DRB11101,HLA-DRB11302,HLA-DRB11501,HLA-DRB30101,HLA-DPA10103-DPB10401,HLA-DPA10103-DPB10201,HLA-DPA10201-DPB10101,HLA-DPA10103-HLA-DPB10301_DPB10401,HLA-DPA10301-DPB10402 ,HLA-DQA10501-DQB10301	16
		RVRLRRLFSIRTNAL	HLA-DRB10101,HLA-DRB10401,HLA-DRB10404,HLA-DRB10405 HLA-DRB10701,HLA-DRB10802,HLA-DRB10901,HLA-DRB11101,HLA-	14

			DRB11302,HLA-DRB11501,HLA-DRB40101,HLA-DRB50101,HLA-DPA10201-DPB10101,HLA-DPA10301-DPB10402	
3	XP_003863 771.1	LLALILLGGIGAVGY	HLA-DRB10101, HLA-DRB10404, HLA-DRB10901, HLA-DRB11501, HLA-DQA10501-DQB10301	5
		YPVYPFLASNAALLN	HLA-DRB10101, HLA-DRB10301, HLA-DRB10401, HLA-DRB10404, HLA-DRB10405, HLA-DRB10701, HLA-DRB10802, HLA-DRB10901, HLA-DRB11101, HLA-DRB11302, HLA-DRB11501, HLA-DRB30101, HLA-DRB50101,HLA-DPA10103-DPB10401, HLA-DPA10103-DPB10201, HLA-DPA10201-DPB10101, HLA-DPA10103-HLA-DPB10301_DPB10401, HLA-DPA10301-DPB10402,HLA-DQA10102-DQB10602,HLA-DQA10401-DQB10402, HLA-DQA10501-DQB10201,HLA-DQA10501-DQB10301	22
		LPSFHAMSAFHSAAK	HLA-DRB10101, HLA-DRB10401, HLA-DRB10404, HLA-DRB10405, HLA-DRB10701, HLA-DRB10901, HLA-DRB11101,HLA-DRB11501, HLA-DRB40101, HLA-DRB50101, HLA-DQA10102-DQB10602, HLA-DQA10501-DQB10301,	12
		NAALLNLIPSLLYRV	HLA-DRB10101,HLA-DRB10301,HLA-DRB10401,HLA-DRB10404,HLA-DRB10405,HLA-DRB10701,HLA-DRB10802,HLA-DRB10901,HLA-DRB11101,HLA-DRB11302,HLA-DRB11501,HLA-DRB40101,HLA-DRB50101,HLA-DPA10103-DPB10401,HLA-DPA10103-DPB10201, HLA-DPA10201-DPB10101,HLA-DPA10103-HLA-DPB10301_DPB10401, HLA-DPA10301-DPB10402, HLA-DQA10102-DQB10602,	19
		VYPFLASNAALLNLI	HLA-DRB10101,HLA-DRB10301,HLA-DRB10401,HLA-DRB10404,HLA-DRB10405,HLA-DRB10701,HLA-DRB10901,HLA-DRB11101,HLA-DRB11302,HLA-DRB11501, HLA-DRB30101, HLA-DRB50101, HLA-DPA10103-DPB10401,HLA-DPA10103-DPB10201,HLA-DPA10201-DPB10101,HLA-DPA10301-DPB10402,HLA-DQA10102-DQB10602, HLA-DQA10501-DQB10201 , HLA-DQA10501-DQB10301	19
		RALLALILLGGIGAV	HLA-DRB10101, HLA-DRB10404, HLA-DRB11501, HLA-DPA10301-DPB10402, HLA-DQA10501-DQB10301	5
		AALLNLIPSLLYRVQ	HLA-DRB10101,HLA-DRB10301,HLA-DRB10401,HLA-DRB10404,HLA-DRB10405,HLA-DRB10701,HLA-DRB10802,HLA-DRB10901,HLA-DRB11101,HLA-DRB11302,HLA-DRB11501,HLA-DRB40101,HLA-DRB50101,HLA-DPA10103-DPB10401,HLA-DPA10103-DPB10201,HLA-DPA10201-DPB10101,HLA-DPA10201-DPB10501,HLA-DPA10103-HLA-DPB10301 _DPB10401, HLA-DPA10301-DPB10402, HLA-DQA10102-DQB10602	20
4	XP_003858 976.1	LQVFTAICASFAHGA	HLA-DRB10101,HLA-DRB10401,HLA-DRB10404,HLA-DRB10405,HLA-DRB10701,HLA-DRB10802,HLA-DRB10901,HLA-DRB11101,HLA-DRB11501,HLA-DRB50101,HLA-DQA10102-DQB10602,HLA-DQA10501-DQB10301	12
		FPFFSGVAPIVASWF	HLA-DRB10101,HLA-DRB10401,HLA-DRB10404,HLA-DRB10405,HLA-DRB10701,HLA-DRB10802,HLA-DRB10901,HLA-DRB11101,HLA-DRB11302,HLA-DQA10102-DQB10602,HLA-DQA10501-DQB10201,HLA-DQA10501-DQB10301,	12
		ERVFRYLQVFTAICA	HLA-DRB10101,HLA-DRB10401,HLA-DRB10404,HLA-DRB10405,HLA-DRB10701,HLA-DRB10802,HLA-DRB11101,HLA-DRB11501,HLA-DRB40101,HLA-DRB50101,HLA-DPA10103-DPB10401,HLA-DPA10103-DPB10201,HLA-DPA10201-DPB10101,HLA-DPA10201-DPB10501,HLA-DPA10103-HLA-DPB10301_DPB10401,HLA-DPA10301-DPB10402,HLA-DQA10102-DQB10602,HLA-DQA10501-DQB10301	18
		KDDFPFFSGVAPIVA	HLA-DRB10101,HLA-DRB10401,HLA-DRB10404,HLA-DRB10405,HLA-DRB10701,HLA-DRB10901,HLA-DRB11101,HLA-DRB11302,HLA-DRB11501,HLA-DPA10103-DPB10201,HLA-DQA10501-DQB10201,HLA-DQA10501-DQB10301	12
		DFPFFSGVAPIVASW	HLA-DRB10101,HLA-DRB10401,HLA-DRB10404,HLA-DRB10405,HLA-DRB10701,HLA-DRB10901,HLA-DRB11101,HLA-DRB11302,HLA-DRB11501,HLA-DQA10102-DQB10602,HLA-DQA10501-DQB10301	11
		LESFFVLFKGASKRL	HLA-DRB10101,HLA-DRB10401,HLA-DRB10404,HLA-DRB10405,HLA-DRB10701,HLA-DRB10901,HLA-DRB11101,HLA-DRB11501,HLA-DRB50101,HLA-DPA10103-DPB10201,HLA-DPA10201-DPB10101,HLA-DPA10201-DPB10501,HLA-DPA10103-HLA-DPB10301_DPB10401,HLA-DPA10301-DPB10402,HLA-DQA10501-DQB10301	15
		FFVLFKGASKRLKWS	HLA-DRB10101,HLA-DRB10401,HLA-DRB10404,HLA-DRB10405,HLA-RB10701,HLA-DRB10901,HLA-DRB11101,HLA-DRB11501,HLA-DRB50101,HLA-DPA10201-DPB10101,HLA-DPA10201-DPB10501,HLA-DPA10301-DPB10402,HLA-DQA10501-DQB10301,	13
		FRYLQVFTAICASFA	HLA-DRB10101,HLA-DRB10401,HLA-DRB10404,HLA-DRB10405, HLA-DRB10701,HLA-DRB10802,HLA-DRB10901,HLA-DRB11101,HLA-	18

			DRB11501,HLA-DRB50101,HLA-DPA10103-DPB10401,HLA-DPA10103-DPB10201,HLA-DPA10201-DPB10101,HLA-DPA10201-DPB10501,HLA-DPA10103-HLA-DPB10301_DPB10401 ,HLA-DPA10301-DPB10402,HLA-DQA10102-DQB10602,HLA-DQA10501-DQB10301	
		SRGFSAELSAALVVS	HLA-DRB10101,HLA-DRB10301,HLA-DRB10401,HLA-DRB10405,HLA-DRB10701,HLA-DRB10901,HLA-DRB11501,HLA-DRB30101,HLA-DRB50101,HLA-DPA10301-DPB10402,HLA-DQA10102-DQB10602,HLA-DQA10301-DQB10302,HLA-DQA10401-DQB10402,HLA-DQA10501-DQB10301	14
		RGFSAELSAALVVSF	HLA-DRB10101,HLA-DRB10401,HLA-DRB10405,HLA-DRB10701,HLA-DRB10901,HLA-DRB11501,HLA-DRB30101,HLA-DRB50101,HLA-DPA10301-DPB10402,HLA-DQA10102-DQB10602,HLA-DQA10301-DQB10302,HLA-DQA10401-DQB10402,HLA-DQA10501-DQB10301	13
5	XP_003857 910.1	LALLIMLYALIATQF	HLA-DRB10101,HLA-DRB10401,HLA-DRB10404,HLA-DRB10405,HLA-DRB10701,HLA-DRB10802,HLA-DRB10901,HLA-DRB11501,HLA-DRB50101,HLA-DPA10103-DPB10201,HLA-DPA10201-DPB10101,HLA-DPA10301-DPB10402,HLA-DQA10101-DQB10501,HLA-DQA10102-DQB10602,HLA-DQA10301-DQB10302,HLA-DQA10401-DQB10402,HLA-DQA10501-DQB10201,HLA-DQA10501-DQB10301	18
		LIMLYALIATQFSDD	HLA-DRB10101,HLA-DRB10401,HLA-DRB10404,HLA-DRB10405,HLA-DRB10701,HLA-DRB10802,HLA-DRB10901,HLA-DRB11101,HLA-DRB11501,HLA-DRB40101,HLA-DRB50101,HLA-DPA10103-DPB10401,HLA-DPA10103-DPB10201,HLA-DPA10201-DPB10101,HLA-DPA10103-HLA-DPB10301_DPB10401,HLA-DPA10301-DPB10402,HLA-DQA10102-DQB10602,HLA-DQA10301-DQB10302,HLA-DQA10401-DQB10402,HLA-DQA10501-DQB10201,HLA-DQA10501-DQB10301	21
		IMLYALIATQFSDDA	HLA-DRB10101,HLA-DRB10401,HLA-DRB10404,HLA-DRB10405,HLA-DRB10701,HLA-DRB10802,HLA-DRB10901,HLA-DRB11101,HLA-DRB11501,HLA-DRB40101,HLA-DRB50101,HLA-DPA10103-DPB10401,HLA-DPA10103-DPB10201,HLA-DPA10201-DPB10101,HLA-DPA10103-HLA-DPB10301_DPB10401 ,HLA-DPA10301-DPB10402,HLA-DQA10102-DQB10602,HLA-DQA10301-DQB10302,HLA-DQA10401-DQB10402,HLA-DQA10501-DQB10201,HLA-DQA10501-DQB10301	21
		VSVLALLIMLYALIA	HLA-DRB10101,HLA-DRB10404,HLA-DRB10405,HLA-DRB11501,HLA-DPA10103-HLA-DPB10301_DPB10401,HLA-DPA10301-DPB10402	6
		VLALLIMLYALIATQ	HLA-DRB10101HLA-DRB10404HLA-DRB10405HLA-DRB11501HLA-DPA10103-DPB10201,HLA-DPA10301-DPB10402,HLA-DQA10102-DQB10602,HLA-DQA10401-DQB10402,HLA-DQA10501-DQB10301	9
6	XP_003858 984.1	VSILRQLLSVTAHTH	HLA-DRB10101,HLA-DRB10401,HLA-DRB10404,HLA-DRB10405,HLA-DRB10701,HLA-DRB10802,HLA-DRB10901,HLA-DRB11101,HLA-DRB11501,HLA-DRB40101,HLA-DRB50101,HLA-DPA10201-DPB10101,HLA-DPA10301-DPB10402,HLA-DQA10102-DQB10602	14
		KVAVSILRQLLSVTA	HLA-DRB10101,HLA-DRB10301,HLA-DRB10401,HLA-DRB10404,HLA-DRB10405,HLA-DRB10701,HLA-DRB10802,HLA-DRB10901,HLA-DRB11101,HLA-DRB11501,HLA-DRB40101,HLA-DRB50101,HLA-DPA10103-DPB10401,HLA-DPA10103-DPB10201,HLA-DPA10201-DPB10101,HLA-DPA10201-DPB10501,HLA-DPA10103-HLA-DPB10301_DPB10401,HLA-DPA10301-DPB10402,HLA-DQA10102-DQB10602	19
		GISFSRAFAANIESA	HLA-DRB10101,HLA-DRB10401,HLA-DRB10404,HLA-DRB10405,HLA-DRB10701,HLA-DRB10802,HLA-DRB10901,HLA-DRB11101,HLA-DRB11302,HLA-DRB11501,HLA-DRB50101,HLA-DQA10102-DQB10602,HLA-DQA10301-DQB10302,HLA-DQA10401-DQB10402,HLA-DQA10501-DQB10301	15
		YQFYHRARSYVIFTT	HLA-DRB10101,HLA-DRB10401,HLA-DRB10404,HLA-DRB10405,HLA-DRB10701,HLA-DRB10901,HLA-DRB11101,HLA-DRB11501,HLA-DRB50101,HLA-DQA10501-DQB10301	10
