# Supplementary figures and images for: Identification of Potential MHC Class-II-Restricted Epitopes Derived from Leishmania donovani Antigens by Reverse Vaccinology and Evaluation of Their CD4+ T-Cell Responsiveness against Visceral Leishmaniasis
Source: Front Immunol. 2017 Dec 14;8:1763. doi: 10.3389/fimmu.2017.01763 (PMC5735068; doi:10.3389/fimmu.2017.01763)

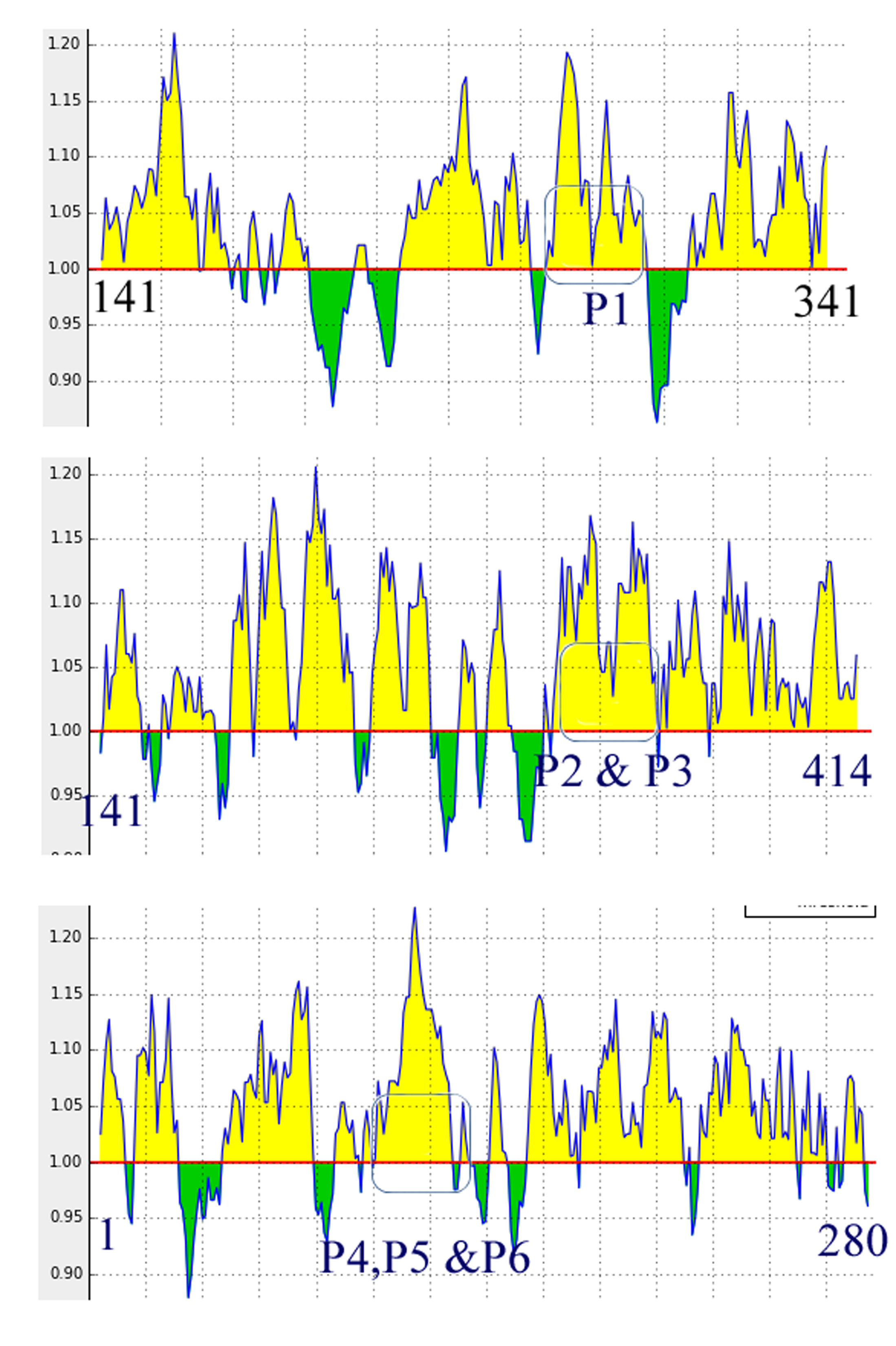

Supplement: Figure S1 — The predicted antigenicity of the optimal set of epitopes by Kolaskar and Tongaonkar Antigenicity method available at IEDB web server. The yellow color represents antigenic regions. [file image_1.tif]
